# Editorial: Supramolecular Nanomaterials for Engineering, Drug Delivery, and Medical Applications

**DOI:** 10.3389/fchem.2020.626468

**Published:** 2020-12-09

**Authors:** Elise Lepeltier, Vincent Levet, Tu Lee, Nathalie Mignet, Jianliang Shen, Hicham Fenniri, Yohann Corvis

**Affiliations:** ^1^Micro et Nanomédecines Translationnelles, MINT, UNIV Angers, Inserm 1066, CNRS, Angers, France; ^2^GSK Vaccines, Rue de l'Institut 89, Rixensart, Belgium; ^3^Department of Chemical and Materials Engineering, National Central University, Taoyuan City, Taiwan; ^4^Université de Paris, CNRS, Inserm, UTCBS, Chemical and Biological Technologies for Health Group (utcbs.cnrs.fr), Faculté de Pharmacie, Paris, France; ^5^School of Ophthalmology & Optometry, School of Biomedical Engineering, Wenzhou Medical University, Wenzhou, China; ^6^Departments of Chemical Engineering, Bioengineering, Chemistry & Chemical Biology, Northeastern University, Boston, MA, United States

**Keywords:** bioinspired nanomaterials, drug delivery and targeting, vaccines production and development, diagnostic agents, cancer treatment

Programmed self-assembly and self-organization of carefully designed molecular monomers (Imai et al., 2018) has been widely explored to engineer stable nanostructures with the desired architecture and unique functionality (Lehn, 2015, 2017). This bottom-up approach could not only overcome design barriers associated with traditional molecular manufacturing at the nanoscale, but it could also endow the desired assemblies with adaptability, tunability, and stimuli-responsiveness due to the dynamic nature of the non-covalent interactions holding the architecture together. Hence, these supramolecular architectures may constitute the basis for novel smart nanomaterials with improved properties such as *in vitro* and *in vivo* physicochemical stability (Park et al., 2007), efficiency *via* drug loading improvement (Ahmed et al., 2019), exogenous environment adaptability (Pedersen et al., 2020), higher safety (Martin et al., 2020), manufacturability (Wren et al., 2020), and may have a broad range of applications with various interfaces, i.e., liquid/liquid (Prevot et al., 2018), solid/liquid (Couillaud et al., 2019), and gas/liquid (Manta et al., 2016; Corvis et al., 2018). Indeed, self-assembled systems (Beingessner et al., 2016; Mohamed et al., 2019) have been developed and widely explored in drug delivery (Chen et al., 2011; Song et al., 2011; Desmaële et al., 2012; Mignet et al., 2012; Al Sabbagh et al., 2020), gene delivery (Manta et al., 2017; Do et al., 2019), biomedical engineering (Sun et al., 2012; Childs et al., 2013; Meng et al., 2013; Puzan et al., 2018; Zhou et al., 2020), medicine (Journeay et al., 2008, 2009; Sun et al., 2014), and diagnostics. This body of work has led to the emergence of the field of supramolecular nanomedicine, which is the focus of this Research Topic for *Frontiers in Chemistry* (Figure 1).

**Figure 1:**
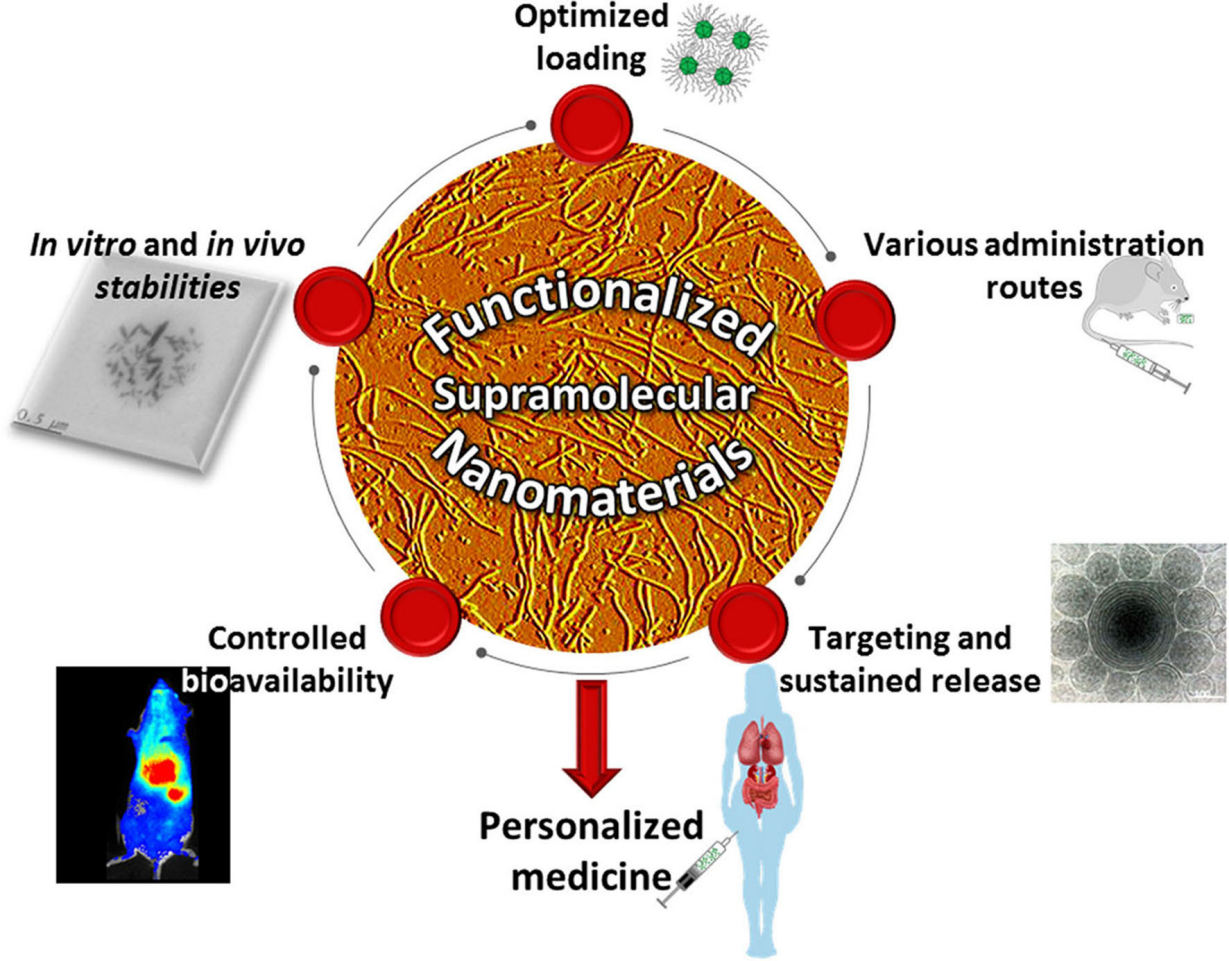
Supramolecular nanomaterials and their applications. Highlight of the fields covered by this Research Topic.

The present Research Topic highlights nanomaterials importance in biological sciences through the supramolecular chemistry prism. Zhang et al. engineered dynamic hydrogels by cross-linking of O-carboxymethyl chitosan with reversibly connected imino-PEGylated dynamers. The double imine chitosan/dynamer and dynamer bonds were considered to provide tangled structures for controlled drug release behavior from the hydrogels. The structural and physical properties of the resulting hydrogels were examined, showing good thermal stability and optimized swelling behavior. When hydrogels with various composition ratios were further developed for the delivery of the anti-cancer drug fluorouracil (5-FU), high drug encapsulation rates up to 97% were obtained. 5-FU *in-vitro* drug-release profiles from the hydrogels showed an initial burst-effect, followed by a slower increase and plateau. All reached a high total release within 12 h, which varied upon the cross-linking level that depended on drug/carrier ratios and gelation temperature, thus demonstrating the potential adjustable sustained release applications of this new type of carrier, notably for topical administration.

However, exogen compounds even at the nanoscale dimension, have the propensity to be recognized by the mononuclear phagocyte system once in the bloodstream, inducing rapid clearance of the antigen from the blood and limiting the sustained release advantage. In order to prolong systemic circulation and to prevent the therapeutic agent from aggregation, opsonization or phagocytosis, nanoparticles have been coated with hydrophilic biocompatible co-polymers since the 1990's (Klibanov et al., 1990; Tan et al., 1993; Fam et al., 2020). Liu et al. are reporting surface functionalized Persistent Luminescence NanoParticles (PLNPs) which have been proved promising nanomaterials for bioimaging applications. For that purpose, poly(N-2-hydroxypropyl)methacrylamide (pHPMA), a hydrophilic polymer widely used in nanomedicine has been intended as an alternative strategy to coat ZnGa_2_O_4_:Cr NPs for *in vivo* imaging. The results demonstrated that the newly designed ZGO NPs can circulate in mouse bloodstream much longer than non-coated ZGO-OH, i.e., at least an hour, without being trapped in the liver or spleen. The new strategy presented allowed to improve nanoparticules already developed by the same group for stability in lysosomal-like medium (Lecuyer et al., 2020) and luminescence efficiency (Maldiney et al., 2014; Teston et al., 2018).

A given nanomaterial obtained by supramolecular interactions may present various potential applications due to the intermolecular non-covalent forces. As outlined by Lu et al., supramolecular materials assembled may present interesting applications in various biomedical fields by integrating multiple interactions of polyphenols, especially tannic acid, a class of plant-derived biocompatible and biodegradable compounds consisting of two or more phenolic units in structure. Starting with the origin of supramolecular assembly, combined with the structural characteristics, biological activity of polyphenol and unique advantages of supramolecular assembly, this review systematically covers the classification of supramolecular assembly, assembly system and applications of tannic acid in, e.g., drug delivery, theranostics for cancer treatment, bone tissue engineering, and biofunctional membrane material. Finally, current and future challenges remaining in this field were put forward. In addition, some traditional or novel assembly techniques were introduced, which also provides some inspiration for the research in this field. Moreover, from a fundamental point of view, the investigation and applications of such dynamic systems could advance our understanding of the design and function of complex chemical and biological systems.

Complex systems can be obtained when ingredients are not in their thermodynamically stable state, e.g., for pharmaceutical liquid forms when drug overall quantity impose by the loading capacity (LC) is higher than its solubility in the same continuous medium, and/or when ingredients that are normally non-miscible are associated together in the same formulation. In such “unstable” systems, excipients are added to kinetically stabilize these ingredients post self-assembly. Ho et al. described the synthesis and characterization of 4 different amphiphilic squalenyl derivatives (i.e., 1 anionic squalenyl derivative, 1 cationic squalenyl-ethanolamine derivative and 2 PEG-squalenyl derivatives of 750 and 3,000 Da, respectively), as well as the physicochemical and biopharmaceutical characterizations of their drug-loaded self-assembled NPs (113–325 nm), in aqueous solution, as drug delivery systems. Seven different model drugs, chosen to exhibit various physicochemical properties (i.e., hydrophobic, hydrophilic, charged) where used with different methods of nanoprecipitation. The best performing methods were determined for each type of formulated system (co-precipitation for hydrophobic compounds, solvent evaporation for hydrophilic compounds, dropping for small and high molecular weight hydrophilic compounds), in relation to the drug properties (molecular weight or logP values). All NP derivatives demonstrated high LC up to 45% w/w, excellent encapsulation efficiency up to 92%, appropriate biocompatibility, adequate colloidal stability in a variety of physiological environments, and sustained release properties exhibiting very low burst-effect and slow controlled-release for at least 24 h. These attributes were achieved thanks to modulated based loading method and loaded NP properties.

Sometime, natural amphiphilic stabilizing agents that are approved by FDA, like silk fibroin, may be preferred as nanocarriers for safety reasons. In their mini review, Ma et al. present the structures of silk fibroin, the controlled transformation of secondary structures, and the formation mechanism of silk fibroin-based nanoparticles and discuss the intrinsic multi-responsibility, surface functionalization, and transgenic modification of these systems for drug delivery. The silk fibroin-based NPs not only have the merits of excellent biocompatibility, but also show the features of multi-responsive systems thanks to stimuli such as pH, hyperthermia, lysosomal enzyme, light, and reactive oxygen species. The multi-responsive phenomenon is helpful for on-demand drug release, reducing systemic side toxicities of some drugs.

Through the non-covalent interactions present in self-assembled therapeutic agents, one of the advantages of smart nanomaterials is that they can be adapted to a specific pathology for personalized medicine purpose. Feng et al. synthesized a novel diblock polymer consisting of polyethylene glycol and poly(glutamic acid (3-(2-nitro-imidazolyl)-propyl)) for hypoxia-responsive polymeric micelle self-assemblies allowing biocompatibility of blank micelles, a drug loading of ~4%, encapsulation efficiency of ~42%, and controlled release properties when loaded with the anticancer agent doxorubicin. The cell experiments ultimately demonstrated that drug-loaded micelles had a stronger apoptosis capacity on tumor cells under hypoxic conditions. All the experiments indicated that latter hypoxia-responsive polymeric micelles have a potential for enhanced cancer treatment since both the rapid and important growth and the relatively insufficient blood supply of the tumor tissues give rise to hypoxic conditions, compared with normal tissues.

However, nanomedicines are not only useful for small organic and inorganic molecules vectorization but also for gene delivery as investigated by Gaillard et al.. They developed dual targeting agents combining the mechanisms of bioactive alkylphospholipids and gene therapies. To achieve this goal, three prodrugs series were prepared based on three cytotoxic alkylphospholipids, namely miltefosine, perifosine, and erufosine. The prodrugs were selectively hydrolyzed at physiological pH to parent cytotoxic drugs. Lipoplexes of the prodrugs with plasmid DNA could transfect cancer cells and produced some enhancement of antiproliferative activity. Therefore, the authors prove that non-classical anti-cancer systems can be also designed by supramolecular formulation of nanomedicines.

To complete the insight into self-assembled nanosystems for treatment and diagnosis applications, supramolecular self-assembled peptide-based vaccines may be considered. Indeed, vaccination is considered, especially these days more than ever due to the coronavirus pandemic, as one of the greatest contributions of science to global health, remarkably through the eradication of great illnesses like smallpox and rinderpest and through its ability to control diseases such as measles and polio (Greenwood, 2014). Since the beginning, vaccination moved from antigens based on attenuated or inactivated viruses to purified proteins and more recently to peptide sub-unit epitopes. The latter are more and more recognized as one of the safest approach relative to control over manufacturing processes, contaminations, undesirable effects and autoimmune responses but sometimes comes at the cost of insufficient biological response. As an alternative, nanovaccines including supramolecular self-assembled peptide-based vaccines may be considered but their development is currently limited by our comprehension of self-assembled nanostructures and immune cells interactions (Negahdaripour et al., 2017; Fadeel, 2019). Advanced engineering of supramolecular assembly composed for instance of cell-penetrating peptides (Bechara and Sagan, 2013; Almeida et al., 2016) as delivery vehicles may help promoting a strong cellular or humoral immune response as they favorize targeted intracellular delivery of antigens. In the present Research Topic, Abudula et al. review the span of strategies applicable to constructing and applying such self-assembled structure as antigens themselves, going from their supramolecular architecture to their multiple application in vaccinology. The mechanisms behind the ability of these peptide-based nanovaccines to generate cell-mediated immunity or humoral response and the aspect of self-adjuvantation are highlighted. Finally, the challenges, clinical translatability and future perspectives are also discussed, considering the great potential of these innovative type of constructs for the future of vaccinology.

Self-assembled systems based on small molecules, block copolymers, dendrimers, peptides, and amphiphiles have been successfully synthesized to achieve a biological, medical, delivery, and/or sensing function(s). Some of these assemblies are designed to be responsive to stimuli such as ultrasound, light, pH, temperature, or specific reagents. Because of their design features and current applications, these supramolecular nanosystems belong to the new and very active field of supramolecular nanomedicine. We thank the 59 co-authors all around the globe which have contributed to this Research Topic. Our main objective is to provide scientists, engineers and clinicians better and newer definition of nanomedicine through the publication of original papers or timely reviews covering synthetically accessible and chemically tunable self-assembled nanosystems endowed with a pharmaceutical, medical, biological, or diagnostic function.

## Dedications

This article is dedicated to Dr. Hai Doan Do, UTCBS' angel who left us too soon.

## Author Contributions

HF and YC have proposed the idea of this Research Topic. All authors contributed to the guest editorial board and the related manuscript has been read and revised by all the authors.

## Conflict of Interest

VL was employed by the GSK group of companies. The remaining authors declare that the research was conducted in the absence of any commercial or financial relationships that could be construed as a potential conflict of interest.
